# The general Assembly of the International Association of Environmental Mutagenesis and Genomics Societies (IAEMGS) in 2017

**DOI:** 10.1186/s41021-018-0097-0

**Published:** 2018-05-07

**Authors:** Takehiko Nohmi, Catarina S. Takahashi, Byung-Mu Lee, Michael F. Fenech, Masami Yamada

**Affiliations:** 1 Biological Safety Research Center, National Institute of Health Sciences, 3-25-26 Tonomachi, Kawasaki-ku, Kawasaki-shi, Kanagawa 210-9501 Japan; 2 Biology Department, Universidade de Sao Paulo, 14090.901 Ribeirao Preto, SP Brazil; 3 College of Pharmacy, Sungkyunkwan University, 300 Cheoncheon-dong, Jangan-gu, Suwon, Gyeonggi-do 440-746 South Korea; 4 Genome Health and Personalised Nutrition Laboratory, CSIRO Health and Biosecurity, PO Box 10041, Adelaide, BC SA 5000 Australia; 5 Department of Applied Chemistry, National Defense Academy of Japan, Hashirimizu 1-10-20, Yokosuka-shi, Kanagawa 239-8686 Japan

**Keywords:** Environment, Genomics, Mutagenesis, Genetic toxicology

## Abstract

The International Association of Environmental Mutagenesis and Genomics Societies (IAEMGS) is a global organization that promotes applied and basic research on environmental mutagenesis and genomics. IAEMGS is composed of 13 national and regional societies of environmental mutagenesis and genomics and the total membership is approximately 4000. IAEMGS convenes the general assembly every four years during each International Conference on Environmental Mutagens (ICEM) for communication among members of participating societies and discussion on future directions. The latest general assembly was held during the 12^th^ ICEM/5^th^ Asian Conference on Environmental Mutagens (ACEM) in November 2017 in Incheon, Korea. This report summarizes the topics and discussions in the general assembly.

IAEMGS is an international organization that promotes research on measures to protect genomic DNA of humans and other organisms from the potential genotoxic and mutagenic effects of environmental chemicals and physical agents [1, 2]. The research that the IAEMGS promotes and supports includes basic research on molecular mechanisms of mutagenesis and DNA repair, molecular epidemiology of humans exposed to environmental mutagens and regulatory genetic toxicology related to test guidelines for genotoxicity testing. IAEMGS was established in 1973 during the 1^st^ International Conference on Environmental Mutagens (ICEM) in Asilomar, USA [3]. The original name of International Association of Environmental Mutagen Societies (IAEMS) was changed to the current name in fall 2013 to emphasize its focus on the study and regulation of genomic effects [4]. IAEMGS is composed of 13 national and regional environmental mutagenesis and genomics societies (EMGS) and environmental mutagen societies (EMS) worldwide. The current membership is approximately 4000. IAEMGS holds the ICEM conference once every four years to provide a means whereby the members exchange the latest knowledge, learn established and novel experimental techniques and organize international collaborations. The 2^nd^ ICEM was held in Edinburgh, UK (1977), followed by the 3^rd^ in Tokyo, Japan (1981); the 4^th^ in Stockholm, Sweden (1985); the 5^th^ in Cleveland, USA (1989); the 6^th^ in Melbourne, Australia (1993); the 7^th^ in Toulouse, France (1997); the 8th in Shizuoka, Japan (2001); the 9^th^ in San Francisco, USA (2005); the 10^th^ Florence, Italy (2009); the 11th in Iguacu, Brazil (2013) and the 12th in Incheon, Korea (2017). The 12^th^ ICEM was held as a joint meeting with the 5^th^ Asian Conference on Environmental Mutagens (ACEM). The general assembly of IAEMGS is convened during each ICEM to provide for communication among member societies and the officers and council of the IAEMGS. The latest general assembly was held on November 13 (Mon), 2017 at Songdo Convensia, the venue of the 12^th^ ICEM/5^th^ ACEM, in Incheon, Korea. This report summarizes the topics and discussions in the assembly.

At first, Takehiko Nohmi, the President of IAEMGS, reported activities of IAEMGS from 2013 to 2017 and current financial and membership status of IAEMGS. IAEMGS supported travel expenses for students at the 4^th^ ACEM in Kolkata, India, in December 2014 and provided a loan to Korean EMS (KEMS), the host society for the 12th ICEM/5th ACEM as a seed money. He pointed out, however, that the support for international conferences related to the research on environmental mutagens was severely restricted because of the financial status of IAEMGS. The assets of IAEMGS have been decreasing steadily from 2009 and the current assets are less than US$5000 except for the loan to KEMS (Fig. 1). The decrease is partly due to reduction of membership of participating societies (Fig. 2) and low annual dues of the member societies (this issue will be discussed later). At the end of 2015, IAEMGS changed the management of their financial asset from AIM to BK Association Management. According to this change, the assets of International Workshop on Genotoxicity Testing (IWGT), which had been also maintained by AIM, were transferred to a UK management company in March 2016. Nohmi stressed that increasing membership and raising more funds are critically important to adequately maintain and improve the activities of IAEMGS. Finally, he acknowledged the Executive Board members of IAEMGS (2013 to 2017), i.e., Michael F. Fenech (MEPSA), the secretary; Byung-Mu Lee (KEMS), the vice-president; Catarina S. Takahashi (MutaGen-Brasil), the vice-president; Masami Yamada (JEMS), the treasurer and Bob Bevans-Kerr (BK Association Management), the Executive Director.

**Fig. 1:**
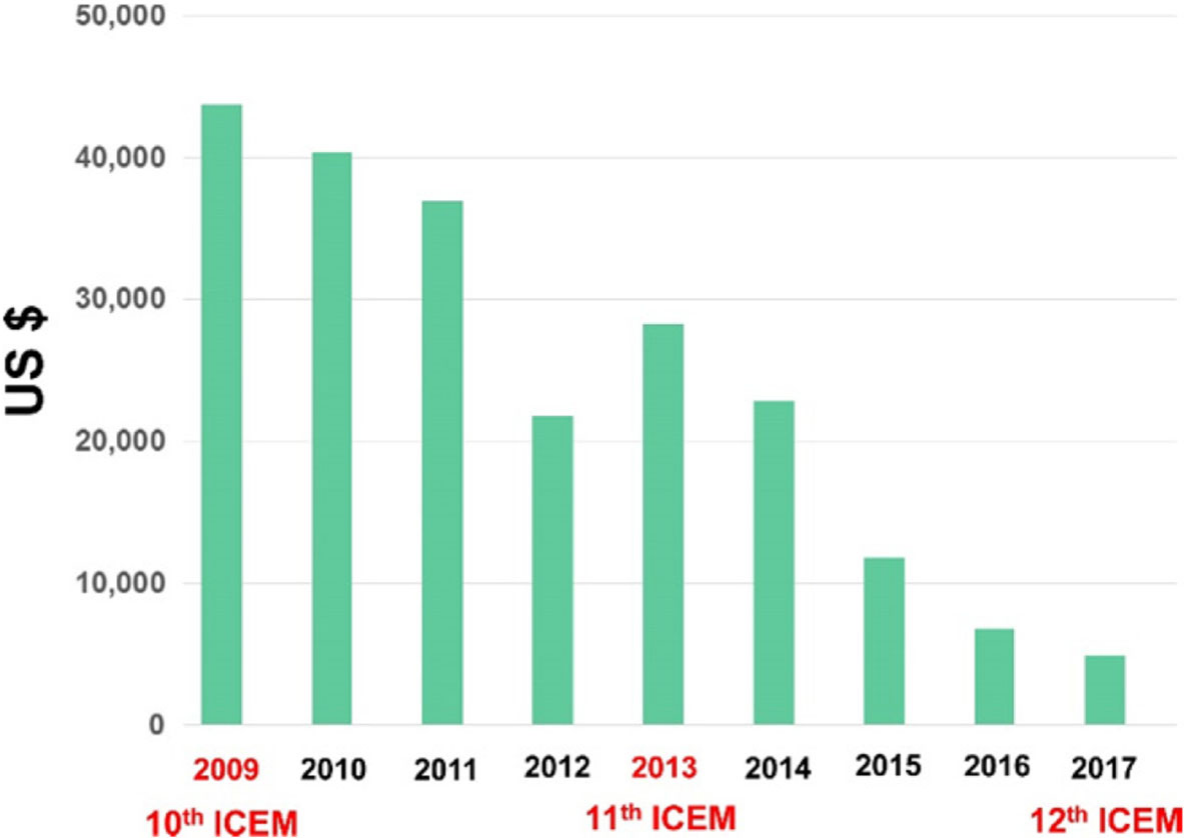
Assets of IAEMGS from 2009 to 2017

**Fig. 2:**
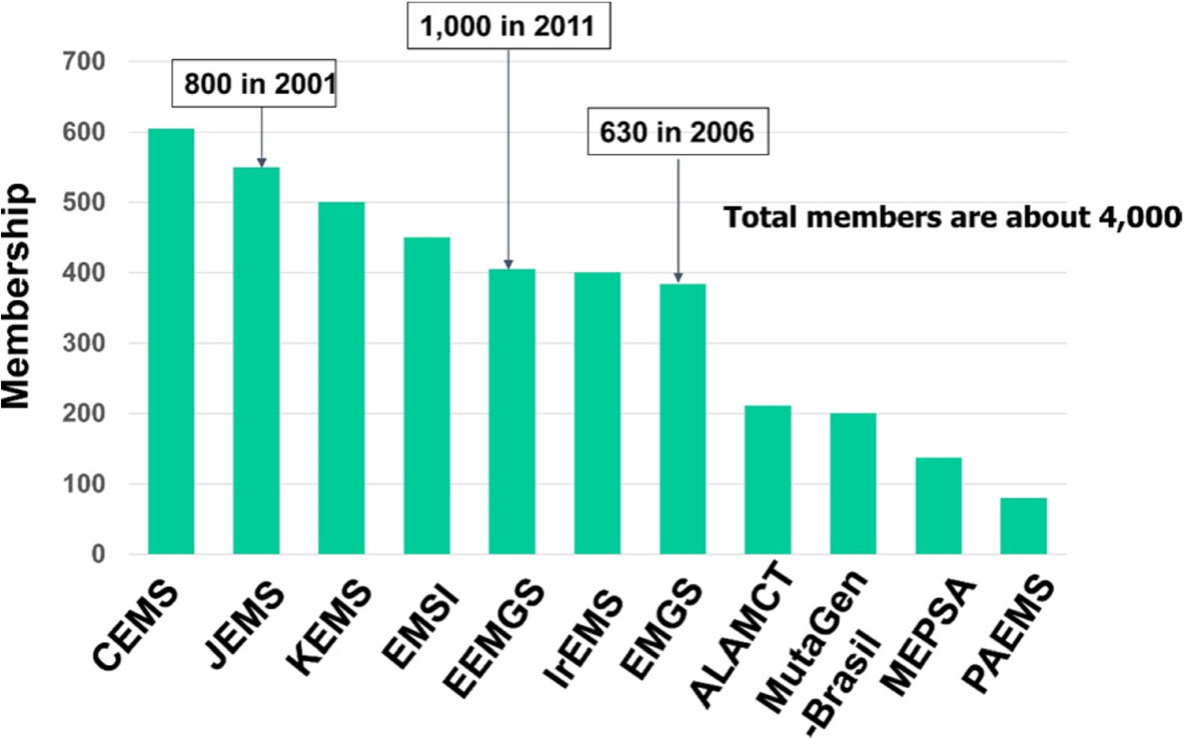
Membership of member societies of IAEMGS. The membership is deduced from the annual due paid by each society in 2015 or 2016. Thai EMS and Philippines EMS are not shown because of no payment in 2015 and 2016. The abbreviations are as follows: CEMS, Chinese EMS; JEMS, The Japanese EMS; KEMS, Korean EMS; EMSI, EMS India; EEMGS, European EMGS; IrEMS, Iranian EMS; EMGS, Environmental Mutagenesis and Genomics Society; ALAMCTA, Asociación Latinoamericana de Mutagénesis, Carcinogénesisy Teratogénesis Ambiental; MutaGen-Brasil, Brazilian Association of Mutagenesis and Environmental Genomics; MEPSA, Molecular and Experimental Pathology Society of Australasia; PAEMS, Pan-African EMS

Secondly, Hoonjeong Kwon, the President of the 12^th^ ICEM/5^th^ ACEM and the President of KEMS/KSOT, reported the status and outcomes of the 12^th^ ICEM/5^th^ ACEM. The conference started in the evening of November 12 (Sun) with an opening ceremony and an opening lecture. Four plenary lectures were set in the morning from Monday through Thursday. Four keynote lectures and two distinguished lectures were presented at noon and late afternoon of Monday and Wednesday. Tuesday afternoon was kept free to allow time for touristic activities or viewing of poster presentations. The conference was scheduled to be closed at around noon of November 16 (Thu) with a closing ceremony. In total, 55 scientific sessions and 473 presentations were made, among which 136 were invited talks and 247 were poster presentations. The Gala dinner was set in the evening on November 15 (Wed). Approximately 700 people attended the conference from 29 countries. About 250 people came from outside of Korea. In conclusion, the conference was very successful in terms of science and a social event.

Thirdly, Paul White, the President of the 13^th^ ICEM, reported the plan for the next ICEM. The 13^th^ ICEM will be held in Ottawa, Canada, from August 28 to September 2, 2021. The venue will be Westin Hotel and Shaw Conference Centre. Since Ottawa is the capital city of Canada and there are many cultural and dining attractions and sports activities in addition to prominent Universities, the conference should be very exciting.

Fourth, Masami Yamada, the Treasurer of IAEMGS, reported the current financial status of IAEMGS. She cautioned that the financial status is deteriorating and that several member societies did not pay the annual dues despite the invoices issued. She also reported the termination of contract with AIM and separation of the assets of IWGT from those of IAEMGS.

Fifth, there was a general discussion regarding the reduction of membership, decreasing assets and name change of EMS to EMGS amongst member societies of IAEMGS. At first, it was questioned whether the reduction of the membership, in particular that of European EMGS (EEMGS), was real. The membership shown in Fig. 2 was based on the annual due that was paid by each EM(G)S in 2015 or 2016. For example, EEMGS has paid US$835 in 2013, which corresponds to 985 membership, but since 2014 it has been paying US$255, which corresponds to 405 membership. Annual due should reflect the actual membership of each society. To increase the assets, it was confirmed and agreed by those present in the assembly that the ICEM is an event that should make a substantial profit for IAEMGS as stated in the Constitution [5] (Bylaws, Article V: ICEM, Section IV) and that ICEM2021 should be designed to make a significant financial contribution to IAEMGS. The current due system was established in 1973 where membership fee is US$0.5 for each of the first 200 members, US$0.75 each for the next 200 members and US$1.00 each for the remaining members (Bylaws, Article IV: Finance). All the participants agreed that the dues should be increased. It was agreed that a key objective of the new IAEMGS Executive Board will be to appropriately increase the annual dues before 2021. With regard to the name change of member societies from EMS to EMGS, both Japanese EMS (JEMS) and Chinese EMS (CEMS) have discussed the issue in their respective societies but both did not support the name change.

Sixth, the newly elected Executive Board members (2017–2021) were introduced as follows: Paul White (EMGS), President; Young-Joon Surh (KEMS), Vice President; Catherine Klein (EMGS), Vice President; Carlos F. M. Menck (MutaGen-Brasil), Secretary; Wilner Martínez-López (ALAMCTA), Treasurer and Bob Bevans-Kerr (BK Association Management), Executive Director.

Finally, a collective photo was taken (Fig. 3) and the assembly was closed.

**Fig. 3:**
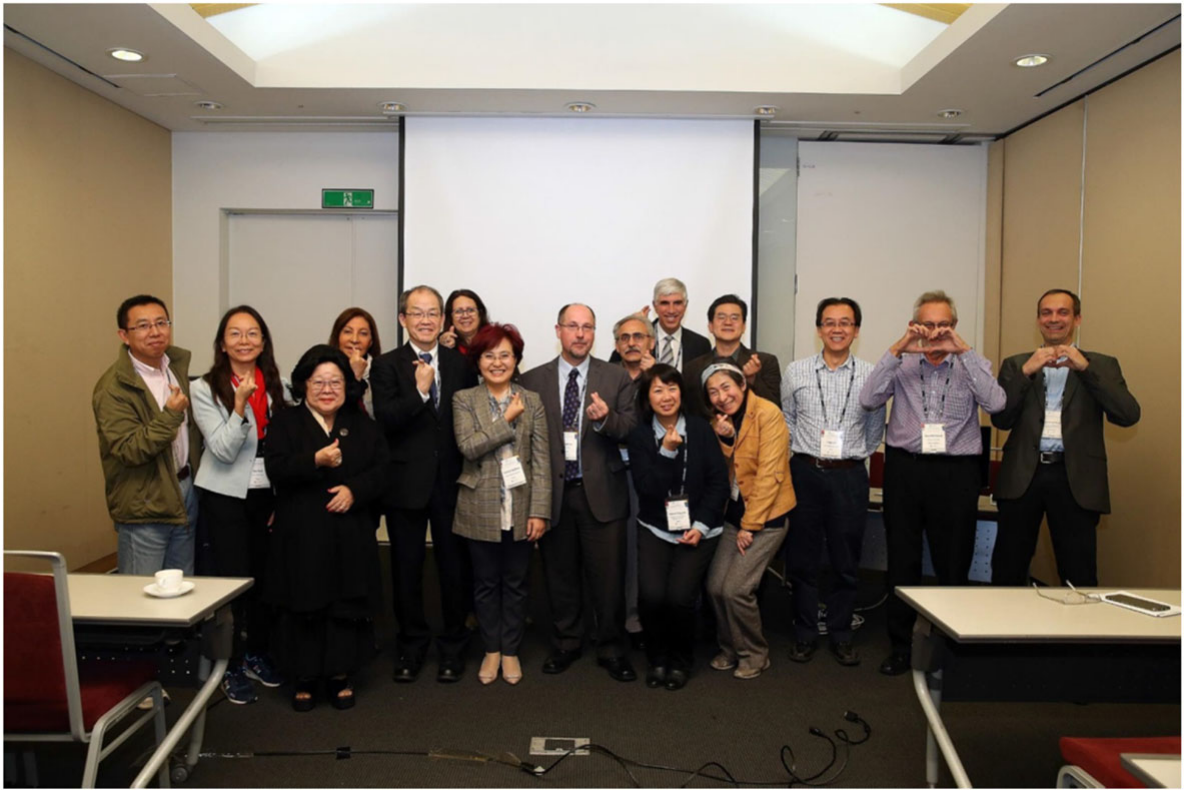
Collective photo of the participants of the General Assembly of IAEMGS during 12^th^ ICEM/5^th^ ACEM on November 13, 2017 in Incheon, Korea. Each one is shaping a heart with hands. From left to right, Jun Yang (CEMS); Xia Huo (CEMS); Cataria S. Takahashi (MutaGen-Brasil); Lucia Regina Ribeiro (MutaGen-Brasil); Takehiko Nohmi (JEMS); Daisy M. F. Salvadori (MutaGen-Brasil); Hoonjeong Kwon (KEMS); Paul White (EMGS); Michael F. Fenech (MEPSA); Yukari Totsuka (JEMS); Stephano Bonassi (EEMGS); Masami Yamada (JEMS); Young Rok Seo (KEMS); Jingbo Pi (CEMS); David Kirkland (EEMGS); Hans-Joerg Martus (EEMGS)

## Abbreviations

ACEM: Asian Conference on Environmental Mutagens
ALAMCT: Asociación Latinoamericana de Mutagénesis, Carcinogénesisy Teratogénesis Ambiental
CEMS: Chinese Environmental Mutagen Society
EEMGS: European Environmental Mutagenesis and Genomics Society
EMGS: Environmental Mutagenesis and Genomics Society
EMS: Environmental Mutagen Society
EMSI: Environmental Mutagen Society in India
IAEMGS: International Association of Environmental Mutagenesis and Genomics Societies
IAEMS: International Association of Environmental Mutagen Societies
ICEM: International Conference on Environmental Mutagens
IrEMS: Iranian Environmental Mutagen Society
IWGT: International Workshop on Genotoxicity Testing
JEMS: Japanese Environmental Mutagen Society
KEMS: Korean Environmental Mutagen Society
MEPSA: Molecular and Experimental Pathology Society of Australasia
MutaGen-Brasil: Brazilian Association of Mutagenesis and Environmental Genomics
PAEMS: Pan-Africanese Environmental Mutagen Society
